# Short‐term high‐fat feeding induces muscle‐type–specific signaling adaptations in skeletal muscle of male rats

**DOI:** 10.14814/phy2.70904

**Published:** 2026-06-15

**Authors:** Arata Tsutaki, Hidetaka Imagita, Asumi Yoshida

**Affiliations:** ^1^ Department of Nutrition and Health Sagami Women's University Sagamihara Kanagawa Japan; ^2^ Present address: Department of Physical Therapy, Faculty of Health and Services Saitama Prefectural University Koshigaya Saitama Japan

**Keywords:** ERK signaling, high‐fat diet, mitochondrial dynamics, muscle‐type specificity, skeletal muscle

## Abstract

Short‐term high‐fat diet (HFD) feeding is used to study metabolic dysregulation, yet many rodent models use extreme fat contents that may not reflect physiological conditions. Skeletal muscle responses to short‐term HFD also vary by muscle type. We tested whether a physiologically relevant HFD induces muscle‐type–specific changes in skeletal muscle signaling and mitochondrial‐related proteins. Male Wistar rats were fed a low‐fat diet (LFD; 10% energy from fat) or HFD (40% energy from fat) for 4 weeks (*n* = 5/group). Soleus and extensor digitorum longus (EDL) muscles were analyzed for OXPHOS complexes, mitochondrial dynamics proteins, and ERK1/2 signaling. HFD increased energy intake and visceral adiposity without changing body weight or muscle mass. In soleus, OXPHOS complex II was reduced, whereas other complexes were preserved. In EDL, phosphorylation of ERK1/2 (Thr202/Tyr204) and Drp1 (Ser616) was reduced without changes in total protein abundance. Thus, short‐term, physiologically relevant HFD feeding induces muscle‐type–specific molecular and signaling adaptations before overt changes in body weight or muscle mass.

## 1 INTRODUCTION

High‐fat diet (HFD) feeding is widely used in rodent models to investigate metabolic dysregulation and cardiometabolic risk. However, the phenotypic outcomes of HFD are highly variable and depend strongly on diet composition, duration of feeding, and the physiological endpoints examined (Buettner et al., 2007; Stott & Marino, 2020). In particular, many experimental HFD paradigms employ extremely high fat contents, often providing ~60% of total energy from fat, which exceeds typical human dietary patterns and may accelerate pathological processes that are not representative of early‐stage dietary lipid excess (Gordon‐Larsen et al., 2021; Speakman, 2019). Consequently, the relevance of such models to physiological or pre‐pathological conditions remains a matter of debate, underscoring the need to examine skeletal muscle responses under more moderate and physiologically relevant fat‐loading conditions.

Skeletal muscle plays a central role in whole‐body metabolic homeostasis, accounting for the majority of insulin‐stimulated glucose uptake and contributing substantially to total energy expenditure (Merz & Thurmond, 2020; Periasamy et al., 2017). As such, the ability of skeletal muscle to maintain metabolic flexibility and functional integrity is critical for buffering excess dietary energy. Previous studies have demonstrated that even relatively short‐term HFD feeding can impair skeletal muscle insulin sensitivity and alter metabolic regulation, indicating that functionally meaningful adaptations can occur within weeks, before overt obesity develops (Turner et al., 2013). Nevertheless, findings regarding mitochondrial‐related adaptations to short‐term HFD remain inconsistent, with reported outcomes differing in magnitude, direction, and muscle specificity.

One important source of this variability is the marked heterogeneity of skeletal muscle. Muscles differ substantially in fiber‐type composition, broadly classified into oxidative slow‐twitch and glycolytic fast‐twitch fibers, which exhibit distinct metabolic and mitochondrial characteristics (Talbot & Maves, 2016). Accumulating evidence indicates that dietary interventions do not affect all skeletal muscles uniformly (Morales et al., 2021). In this context, a previous study (Leduc‐Gaudet et al., 2018) demonstrated that short‐term dietary manipulations elicit divergent mitochondrial and metabolic responses between oxidative and fast‐twitch muscles, even when systemic phenotypes are similar. This work highlighted that muscle‐type–specific analyses are essential for understanding early dietary adaptations, rather than assuming a uniform skeletal muscle response.

Consistent with this view, more recent work has further emphasized that the effects of short‐term high‐fat feeding on skeletal muscle mitochondria are not uniform and can vary across studies and, in some cases, yield seemingly discordant results. Ehrlicher et al. (2021) reported that mitochondrial and metabolic responses to short‐term HFD vary depending on experimental context, including the muscle examined and the duration of feeding, reinforcing the notion that early dietary adaptation is complex and not adequately captured by single‐muscle or single‐endpoint analyses. Together, these findings suggest that discrepancies in the literature may reflect genuine muscle‐type–dependent and time‐dependent responses rather than experimental inconsistency.

Mitochondria are highly dynamic organelles that continuously undergo fusion and fission, processes collectively referred to as mitochondrial dynamics. These processes are increasingly recognized as integral to mitochondrial quality control and metabolic adaptation in skeletal muscle (Drake et al., 2016; Romanello & Sandri, 2015). Importantly, mitochondrial dynamics are not merely secondary consequences of metabolic state but can actively shape muscle phenotype (Liesa & Shirihai, 2013). Recent studies have suggested that mitochondrial fission is involved in the development and functional characteristics of fast‐twitch oxidative muscle fibers, implying that fission‐related signaling pathways may contribute to muscle‐type–specific adaptations (Yasuda et al., 2023). These observations raise the possibility that dietary interventions could differentially modulate mitochondrial dynamics signaling in oxidative versus fast‐twitch muscles.

Dynamin‐related protein 1 (Drp1) is a central regulator of mitochondrial fission, and its activity is modulated by post‐translational modifications, including phosphorylation at Ser616 (corresponding to Ser585 in rodents in some reports) (Taguchi et al., 2007). Phosphorylation at this site is often interpreted as promoting fission‐related activity, although its functional consequences may depend on cellular context. Importantly, upstream kinase pathways can regulate Drp1 Ser616 phosphorylation. In several experimental systems, activation of the MEK/ERK pathway has been linked to increased Drp1 Ser616 phosphorylation, whereas inhibition of ERK signaling reduces phosphorylation at this site (Kashatus et al., 2015; Kitamura et al., 2017). ERK‐dependent regulation of Drp1 Ser616 has also been reported in muscle‐related cellular contexts (Fealy et al., 2014), supporting the plausibility that ERK–Drp1 signaling contributes to skeletal muscle adaptation. Despite this mechanistic framework, it remains unclear whether short‐term, physiologically relevant high‐fat feeding alters ERK signaling and Drp1 phosphorylation in vivo in a muscle‐type–specific manner.

In parallel with signaling regulation, oxidative phosphorylation (OXPHOS)–related proteins provide a proxy for mitochondrial respiratory chain abundance (Larsen et al., 2012). While prolonged HFD feeding is often associated with broad changes in mitochondrial content or function (Morales et al., 2021), the extent to which short‐term HFD induces early remodeling of OXPHOS components remains unresolved (Ehrlicher et al., 2021; Leduc‐Gaudet et al., 2018). Discriminating between early signaling adaptations and later structural remodeling of mitochondrial proteins is therefore essential for understanding the progression of dietary fat–induced muscle adaptation.

Based on these considerations, the aim of the present study was to determine whether a short‐term, physiologically relevant high‐fat diet (40% of total energy from fat) induces muscle‐type–specific alterations in skeletal muscle signaling and mitochondrial‐related proteins. We focused on the oxidative soleus and the fast‐twitch extensor digitorum longus (EDL) muscles and examined ERK1/2 signaling, phosphorylation of Drp1 at Ser616, and the expression of key mitochondrial fusion and fission proteins, together with OXPHOS complex abundance. By analyzing these endpoints in parallel under conditions where body weight and muscle mass remain largely unchanged, we sought to identify early, muscle‐specific molecular adaptations rather than definitive functional consequences during dietary fat exposure.

## 2 MATERIALS AND METHODS

### 2.1 Ethical approval

All experimental procedures were approved by the Animal Experiment Ethics Committee of Kyushu Sangyo University (Approval No. 2021‐002).

### 2.2 Animals and housing

Male Wistar rats (10 weeks old) were obtained from CLEA Japan (Fukuoka, Japan). Animals were housed in temperature‐ and humidity‐controlled facilities under a 12‐h light/dark cycle and had free access to food and water throughout the experimental period.

### 2.3 Experimental design and diets

After a one‐week acclimation period during which all animals were fed a standard chow diet (CE‐2; CLEA Japan), rats were assigned to custom‐formulated experimental diets. Diet formulation and preparation were commissioned to CLEA Japan.

The standard CE‐2 chow provides approximately 12%–13% of total energy from fat; however, this diet was used exclusively during the acclimation period and not during the experimental intervention. During the intervention period, animals received either a high‐fat diet (HFD), providing 40% of total energy from fat, or a low‐fat diet (LFD), providing 10% of total energy from fat. Given the modest difference in fat content between the LFD and the standard CE‐2 chow, CE‐2 feeding was considered appropriate for baseline standardization. Animals were allocated to experimental groups based on body weight measured at the end of the acclimation period to minimize intergroup differences. The macronutrient composition and energy density of the standard chow (CE‐2) and the experimental diets are summarized in Table 1.

**TABLE 1:** Macronutrient composition and energy density of experimental diets.

Parameter	CE‐2	LFD	HFD
Protein (kcal%)	29	20	20
Fat (kcal%)	13	10	40
Carbohydrate (kcal%)	58	70	40
Energy density (kcal/g)	3.42	3.6	4.24
Primary fat source	‐	Cocoa butter	Cocoa butter
Diet formulation	Standard chow	Custom (CLEA Japan)	Custom (CLEA Japan)

*Note*: Values are expressed as kcal% unless otherwise indicated. CE‐2 values are based on the average nutrient analysis provided by CLEA Japan. LFD and HFD were custom‐formulated by CLEA Japan by adjusting the relative proportions of carbohydrate and fat while maintaining protein content at 20% of total energy. Macronutrient composition and energy density were calculated based on food composition database values; measured values may vary slightly depending on the production lot.

### 2.4 Tissue and blood collection

At the end of the feeding period, rats were fasted overnight and anesthetized with 2.5% isoflurane. Blood was collected, and soleus and extensor digitorum longus (EDL) muscles as well as intra‐abdominal adipose tissues were excised and weighed. Muscle weights were averaged between left and right sides and normalized to body weight. Following tissue collection, animals were euthanized by exsanguination via the abdominal aorta. All tissue samples were immediately frozen in liquid nitrogen and stored at −80°C until further analysis.

### 2.5 Plasma biochemical assays

Plasma glucose and total cholesterol concentrations were measured using commercially available assay kits (LabAssay Glucose (cat. no. 291‐94001) and LabAssay Total Cholesterol (cat. no. 293‐93601); Fujifilm Wako, Osaka, Japan) according to the manufacturer's instructions. Absorbance was measured using a Multiskan Sky microplate reader (Thermo Fisher Scientific, Waltham, MA, USA).

### 2.6 Western blotting

For Western blot analysis, 50–100 mg of frozen skeletal muscle tissue from the right hindlimb was used. Whole muscle tissues were homogenized on ice using a pestle homogenizer in ice‐cold RIPA buffer (cat. no. 9806, Cell Signaling Technology [CST], Danvers, MA, USA) supplemented with protease and phosphatase inhibitor cocktails (cat. no. 5872, CST). Homogenates were centrifuged at 14,000×*g* for 10 min at 4°C, and the supernatants were collected.

Protein concentrations were determined using a bicinchoninic acid (BCA) protein assay kit (cat. no. 06385‐00, Nacalai Tesque, Kyoto, Japan). Equal amounts of protein were mixed with 3× loading buffer. Samples were boiled at 95°C for 5 min unless otherwise specified. For immunoblotting with the total OXPHOS antibody cocktail (cat. no. ab110413, Abcam), samples were prepared without boiling, in accordance with the manufacturer's recommendation. Fifteen to twenty micrograms of protein per sample were separated on stain‐free TGX gels (Bio‐Rad, Hercules, CA, USA) and transferred onto PVDF (cat. no. 1704272 or 1704273, Bio‐Rad) membranes using a Trans‐Blot Turbo transfer system (Bio‐Rad).

Membranes were blocked with Bullet Blocking One (cat. no. 13779‐01, Nacalai Tesque) for 5 min at room temperature and washed with TBST. Membranes were incubated overnight at 4°C with primary antibodies diluted in TBST containing 5% Bullet Blocking One. After washing, membranes were incubated with HRP‐linked secondary antibodies (1:2500) for 60 min at room temperature. Immunoreactive bands were visualized by enhanced chemiluminescence (ECL) substrate (Clarity Western ECL Substrate, cat. no. 1705061, Bio‐Rad). For p‐Drp1 (Ser616), Clarity Max Western ECL Substrate (cat. no. 1705062, Bio‐Rad) was used to enhance detection. Signals were captured with a ChemiDoc XRS+ imaging system (Bio‐Rad). Blotted protein signals were normalized to total protein loaded per lane using stain‐free imaging. Total protein was quantified from the stain‐free total protein image acquired with the ChemiDoc XRS+ system (Bio‐Rad) using Image Lab software (Bio‐Rad) by integrating the signal intensity across the entire lane (excluding the molecular weight marker region). The resulting lane total protein signal was used to normalize the intensity of each target band for each sample. Normalization was performed within each blot to account for variability in transfer efficiency and immunodetection. For all targets, exposure times were selected to ensure signals fell within the linear range of detection. When non‐adjacent lanes were presented, this was explicitly indicated in the figure legends.

### 2.7 Antibodies

Drp1 (1:2000, cat. no. 8570, CST); phospho‐Drp1 (Ser616) (1:2000, cat. no. 4494, CST); Fis1 (1:2000, cat. no. 10956‐1‐AP, Proteintech, Tokyo, Japan); Mfn1 (1:2000, cat. no. 13798‐1‐AP, Proteintech); Mfn2 (1:2000, cat. no. 12186‐1‐AP, Proteintech); Opa1 (1:2000, cat. no. 80471, CST); ERK1/2 (1:2000, cat. no. 9102, CST); phospho‐ERK1/2 (Thr202/Tyr204) (1:2000, cat. no. 9101, CST); total OXPHOS antibody cocktail (1:2000, cat. no. ab110413, Abcam, Cambridge, UK). HRP‐linked secondary antibodies were goat anti‐rabbit IgG (cat. no. 7074, CST; 1:2500) and goat anti‐mouse IgG (cat. no. 7076, CST; 1:2500). The anti‐mouse secondary antibody was used only for the OXPHOS antibody cocktail, whereas the anti‐rabbit secondary antibody was used for all other targets.

### 2.8 Statistical analysis

All data are presented as mean ± standard deviation (SD), with individual data points shown when applicable. Between‐group comparisons (LFD vs. HFD) were performed using two‐tailed Welch's *t*‐test in GraphPad Prism (subscription version; GraphPad Software, San Diego, CA, USA). Effect sizes were calculated as Hedges' g in Microsoft Excel and are reported as absolute values. Given the limited sample size and the exploratory nature of the study, inferences were based on the direction and consistency of effects across related outcomes together with their physiological plausibility; *p* values were interpreted as complementary evidence, and results near conventional thresholds were treated cautiously. Graphs were generated using GraphPad Prism.

## 3 RESULTS

### 3.1 Whole‐body phenotype, intake parameters, tissue weights, and plasma indices

**FIGURE 1:**
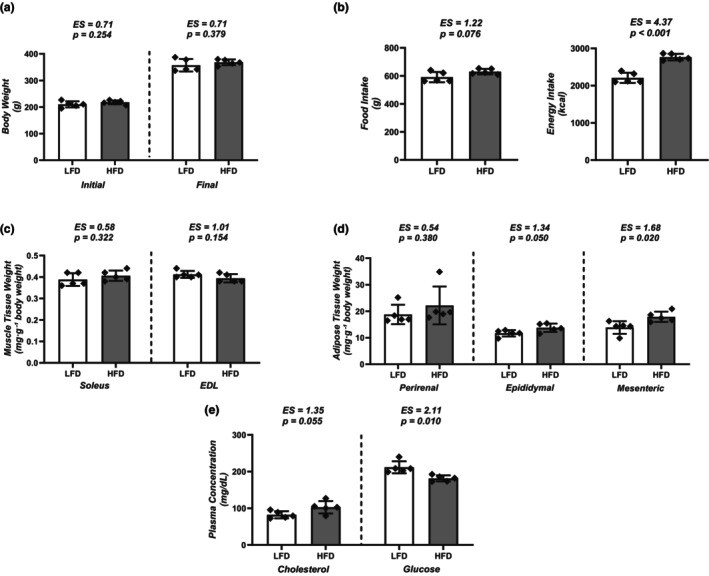
Short‐term high‐fat diet does not alter body weight but increases energy intake and visceral adiposity. Male Wistar rats were fed a low‐fat diet (LFD) or high‐fat diet (HFD) for 4 weeks. Body weight at the beginning and end of the experimental period, total food intake, and total energy intake were assessed cumulatively over 4 weeks. Weights of the soleus and extensor digitorum longus (EDL) muscles (averaged between left and right sides and normalized to body weight), intra‐abdominal adipose tissues (perirenal, epididymal, and mesenteric fat), and plasma concentrations of total cholesterol and glucose were measured. Data are presented as mean ± SD. Statistical comparisons between groups were performed using Welch's *t*‐test, and effect sizes were calculated as Hedges' g. Effect sizes are reported as absolute Hedges' g.

The effects of a short‐term high‐fat diet on whole‐body phenotype, intake parameters, tissue weights, and plasma biochemical indices were evaluated (Figure 1). Body weight did not differ between the LFD and HFD groups at either baseline (ES = 0.71, *p* = 0.254) or the end of the intervention (ES = 0.71, *p* = 0.379) (Figure 1a). Total food intake tended to be higher in the HFD group but did not reach statistical significance (ES = 1.22, *p* = 0.076), whereas total energy intake was markedly higher in the HFD group (ES = 4.37, *p* < 0.001) (Figure 1b). Body weight–normalized muscle weights were not significantly different between groups for either the soleus (ES = 0.58, *p* = 0.322) or the extensor digitorum longus (EDL) muscle (ES = 1.01, *p* = 0.154) (Figure 1c). Among intra‐abdominal adipose depots, perirenal fat did not differ between groups (ES = 0.54, *p* = 0.380), while epididymal fat showed a borderline increase in the HFD group (ES = 1.34, *p* = 0.050) and mesenteric fat was significantly higher in the HFD group (ES = 1.68, *p* = 0.020) (Figure 1d). For plasma parameters, total cholesterol tended to be higher in the HFD group (ES = 1.35, *p* = 0.055), whereas plasma glucose was significantly lower in the HFD group (ES = 2.11, *p* = 0.010) (Figure 1e).

### 3.2 OXPHOS‐related protein expression in soleus and EDL

**FIGURE 2:**
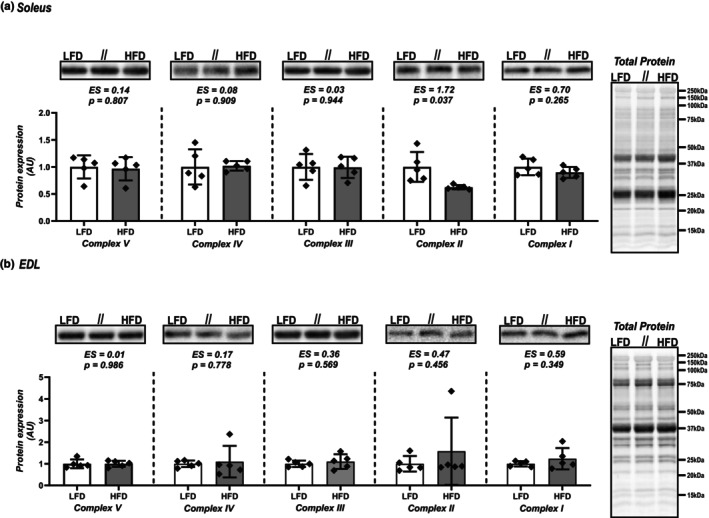
Effects of a short‐term high‐fat diet on oxidative phosphorylation–related protein expression in skeletal muscle. Protein expression of oxidative phosphorylation (OXPHOS) complexes (Complexes I–V) was evaluated in the soleus (A) and EDL (B) muscles by western blotting. Representative blots are shown. Signals were normalized to total protein (stain‐free). LFD and HFD samples were run on the same gel but in non‐adjacent lanes. Effect sizes (Hedges' g) and *p* values (Welch's *t*‐test) are shown for each comparison. Effect sizes are reported as absolute Hedges' g. Full, uncropped blots with molecular weight markers and lane annotations are provided in Figure S1.

To determine whether short‐term high‐fat feeding alters mitochondrial respiratory chain protein abundance, oxidative phosphorylation (OXPHOS)–related protein expression was assessed in the soleus and EDL muscles (Figure 2). In the soleus, no significant differences were observed between the LFD and HFD groups for Complex V (ES = 0.14, *p* = 0.807), Complex IV (ES = 0.08, *p* = 0.909), Complex III (ES = 0.03, *p* = 0.944), or Complex I (ES = 0.70, *p* = 0.265); in contrast, Complex II was significantly lower in the HFD group (ES = 1.72, *p* = 0.037) (Figure 2a). In the EDL, expression levels of OXPHOS complexes were not significantly different between groups for Complex V (ES = 0.01, *p* = 0.986), Complex IV (ES = 0.17, *p* = 0.778), Complex III (ES = 0.36, *p* = 0.569), Complex II (ES = 0.47, *p* = 0.456), and Complex I (ES = 0.59, *p* = 0.349) (Figure 2b).

### 3.3 Mitochondrial fusion‐related proteins

**FIGURE 3:**
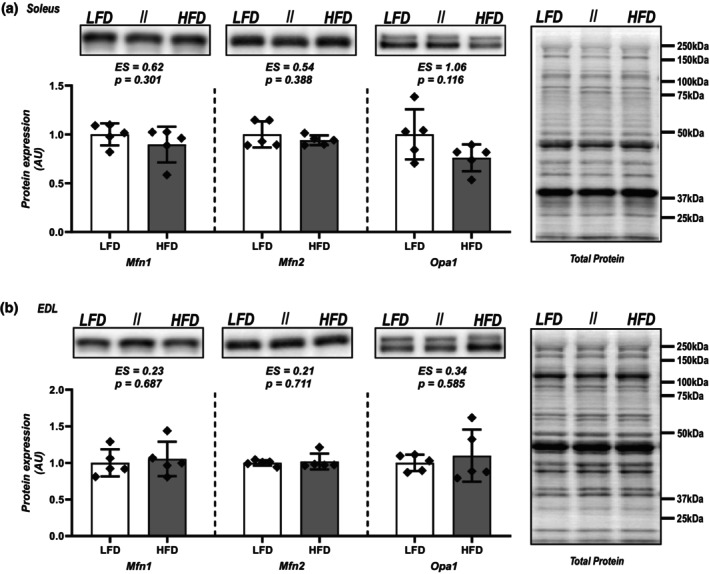
Effects of a short‐term high‐fat diet on mitochondrial fusion–related protein expression in skeletal muscle. Protein expression of mitochondrial fusion–related proteins, mitofusin 1 (Mfn1), mitofusin 2 (Mfn2), and optic atrophy 1 (Opa1) was assessed in the soleus (A) and EDL (B) muscles. Protein expression levels were normalized to total protein determined by stain‐free imaging. LFD and HFD samples were run on the same gel but in non‐adjacent lanes. Effect sizes (Hedges' g) and *p* values (Welch's *t*‐test) are shown for each comparison. Effect sizes are reported as absolute Hedges' g. Full, uncropped blots with molecular weight markers and lane annotations are provided in Figure S2.

Next, mitochondrial fusion–related proteins were examined (Figure 3). In the soleus, no significant differences were observed between the LFD and HFD groups for mitofusin 1 (Mfn1; ES = 0.62, *p* = 0.301) or mitofusin 2 (Mfn2; ES = 0.54, *p* = 0.388). Optic atrophy 1 (Opa1) showed a moderate‐to‐large effect size (ES = 1.06) but did not reach statistical significance (*p* = 0.116) (Figure 3a). In the EDL, expression levels of Mfn1 (ES = 0.23, *p* = 0.687), Mfn2 (ES = 0.21, *p* = 0.711), and Opa1 (ES = 0.34, *p* = 0.585) were not significantly different between the LFD and HFD groups (Figure 3b).

### 3.4 Mitochondrial fission‐related proteins and Drp1 phosphorylation

**FIGURE 4:**
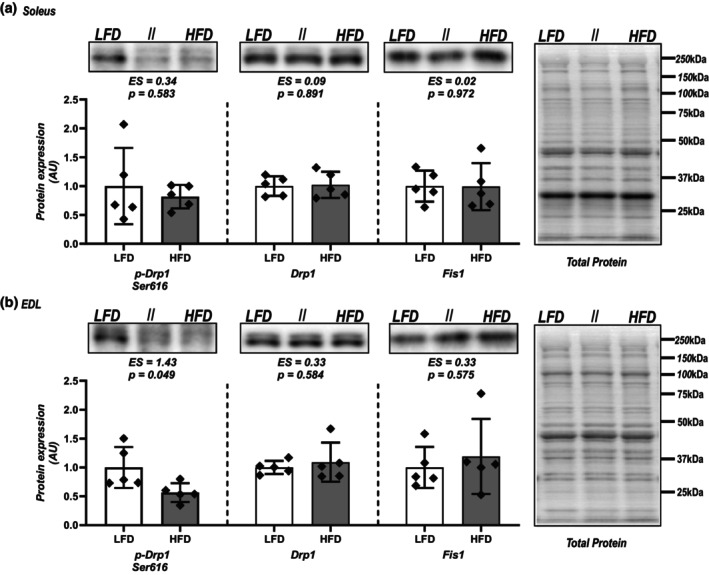
Effects of a short‐term high‐fat diet on mitochondrial fission–related protein expression and phosphorylation in skeletal muscle. Protein expression of mitochondrial fission–related proteins, dynamin‐related protein 1 (Drp1) and fission protein 1 (Fis1), as well as phosphorylation of Drp1 at Ser616, was evaluated in the soleus (A) and EDL (B) muscles. Protein expression levels were normalized to total protein determined by stain‐free imaging. LFD and HFD samples were run on the same gel but in non‐adjacent lanes. Effect sizes (Hedges' g) and *p* values (Welch's *t*‐test) are shown for each comparison. Effect sizes are reported as absolute Hedges' g. Full, uncropped blots with molecular weight markers and lane annotations are provided in Figure S3.

Mitochondrial fission–related protein expression and Drp1 phosphorylation were then assessed (Figure 4). In the soleus, phosphorylated Drp1 at Ser616 was not significantly different between groups (ES = 0.34, *p* = 0.583). Total Drp1 (ES = 0.09, *p* = 0.891) and fission protein 1 (Fis1; ES = 0.02, *p* = 0.972) were also comparable between groups (Figure 4a). In contrast, in the EDL, p‐Drp1 (Ser616) was lower in the HFD group with a large effect size and a borderline p value (ES = 1.43, *p* = 0.049), whereas total Drp1 (ES = 0.33, *p* = 0.584) and Fis1 (ES = 0.33, *p* = 0.575) did not differ between groups (Figure 4b).

### 3.5 ERK1/2 signaling

**FIGURE 5:**
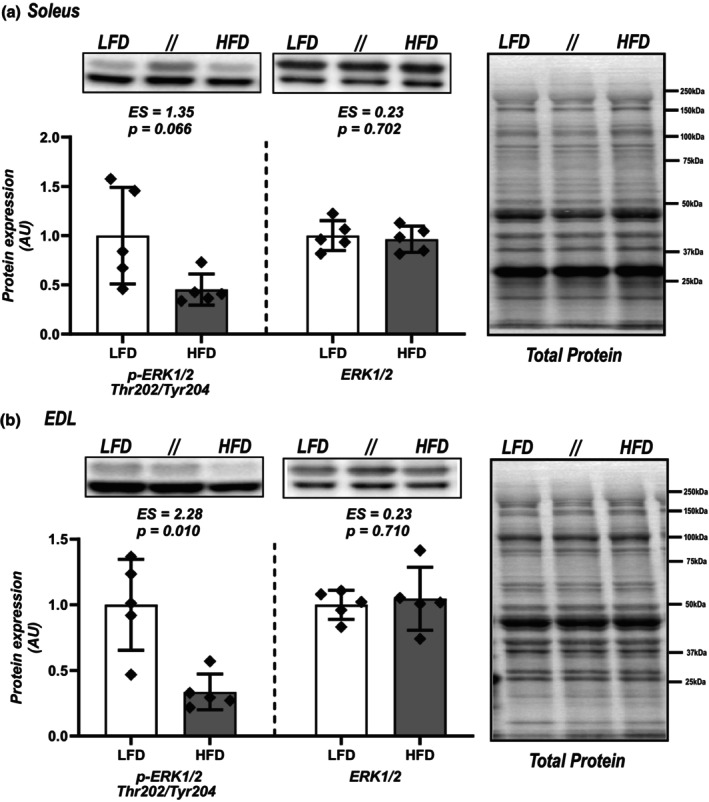
Effects of a short‐term high‐fat diet on ERK1/2 signaling in skeletal muscle. Protein expression of extracellular signal–regulated kinase 1/2 (ERK1/2) and phosphorylated ERK1/2 was assessed in the soleus (A) and EDL (B) muscles by Western blotting. Protein expression levels were normalized to total protein determined by stain‐free imaging. LFD and HFD samples were run on the same gel but in non‐adjacent lanes. Effect sizes (Hedges' g) and *p* values (Welch's *t*‐test) are shown for each comparison. Effect sizes are reported as absolute Hedges' g. Full, uncropped blots with molecular weight markers and lane annotations are provided in Figure S4.

Finally, ERK1/2 signaling was assessed (Figure 5). In the soleus, phosphorylated ERK1/2 at Thr202/Tyr204 (p‐ERK1/2) tended to be lower in the HFD group, showing a large effect size without reaching statistical significance (ES = 1.35, *p* = 0.066), while total ERK1/2 expression was comparable between groups (ES = 0.23, *p* = 0.702) (Figure 5a). In the EDL, p‐ERK1/2 (Thr202/Tyr204) was significantly lower in the HFD group with a very large effect size (ES = 2.28, *p* = 0.010), whereas total ERK1/2 expression did not differ between groups (ES = 0.23, *p* = 0.710) (Figure 5b).

## 4 DISCUSSION

In the present study, we examined the impact of a short‐term (4‐week feeding), physiologically relevant high‐fat diet (40% of total energy from fat) on skeletal muscle signaling and mitochondrial protein profiles in rats. The macronutrient composition and energy density of the experimental diets are summarized in Table 1. The key observation is that this dietary intervention increased energy intake and selectively expanded intra‐abdominal adipose depots (epididymal and mesenteric fat) without altering body weight or body weight–normalized soleus and EDL muscle masses. Importantly, under this condition—that is, prior to overt weight gain—the molecular responses were muscle‐type specific: OXPHOS‐related proteins were largely preserved, except for a selective reduction of Complex II in the soleus, whereas phosphorylation states of ERK1/2 and Drp1 were decreased in the EDL despite unchanged total protein abundance of ERK1/2 and Drp1. This muscle‐type–specific pattern is consistent with prior evidence showing that short‐term high‐fat feeding can elicit divergent mitochondrial and metabolic responses between oxidative and fast‐twitch muscles (Andrich et al., 2018; Leduc‐Gaudet et al., 2018).

A notable finding is the dissociation between “protein abundance” and “phospho‐state” changes, particularly in the fast‐twitch EDL. In the EDL, p‐ERK1/2 (Thr202/Tyr204) was significantly reduced with a very large effect size, and p‐Drp1 (Ser616) showed a concordant decrease with a large effect size that reached borderline statistical significance and should therefore be interpreted cautiously given the limited sample size. In contrast, total ERK1/2 and total Drp1 protein levels were unaltered. This pattern is consistent with a regulatory mode in which short‐term dietary fat exposure shifts signaling activity rather than remodeling protein abundance, a concept that aligns with prior reports of early fiber‐type–dependent molecular responses to short‐term high‐fat feeding. Mechanistically, we refrain from asserting a causal chain; however, multiple experimental systems indicate that ERK activity can modulate Drp1 Ser616 phosphorylation and thereby mitochondrial fission propensity (Kashatus et al., 2015; Kitamura et al., 2017). For example, ERK1/2‐dependent increases in Drp1 Ser616 phosphorylation have been reported in several contexts, including growth factor–driven signaling and stress‐responsive models, supporting biological plausibility for an ERK–Drp1 linkage (Fealy et al., 2014). Our data are therefore consistent with an ERK–Drp1 signaling relationship, but the present design does not establish causality or demonstrate mitochondrial fission‐related structural change.

The selective reduction of Complex II in the soleus, in the absence of broad OXPHOS remodeling, suggests an early and targeted adaptation in oxidative muscle. Complex II (succinate dehydrogenase) uniquely links the tricarboxylic acid cycle and the electron transport chain; therefore, changes in Complex II abundance may reflect a pathway‐selective adjustment of succinate‐linked oxidative metabolism rather than generalized OXPHOS remodeling. One possible metabolic implication is that this response may help adjust succinate‐linked electron entry into the respiratory chain under increased lipid availability, thereby contributing to mitochondrial metabolic flexibility during early dietary adaptation. Together with the lack of changes in other OXPHOS complexes and the use of total‐protein normalization, this finding argues against a generalized loss of mitochondrial content. At present, we interpret this finding as evidence of a pathway‐selective molecular adaptation rather than as evidence of broad mitochondrial dysfunction. This interpretation is consistent with prior evidence that short‐term HFD can elicit heterogeneous mitochondrial responses between soleus and EDL muscles in young rats after 14 days (Leduc‐Gaudet et al., 2018). However, because substrate‐specific mitochondrial respiration, reactive oxygen species production, and upstream regulatory pathways were not directly assessed, the metabolic significance and mechanistic basis of this selective Complex II reduction remain speculative and require further investigation.

Our results also support the concept that early changes in skeletal muscle signaling can emerge during short‐term high‐fat dietary intake before overt obesity develops, although the direction and magnitude of changes may depend on diet composition, duration, and sampling conditions (Ehrlicher et al., 2021; Turner et al., 2013). Several studies using ~4‐week high‐fat feeding have documented alterations in skeletal muscle metabolic regulation and insulin‐related outcomes (Boon et al., 2015; Turner et al., 2013), indicating that meaningful molecular adaptations can occur on this timescale. In the present dataset, plasma glucose was lower in the HFD group under fasting conditions; we interpret this cautiously and view it as evidence that systemic carbohydrate–lipid handling is already altered, rather than as proof of improved glucose homeostasis. Future studies incorporating insulin measurements, glucose tolerance testing, and tissue‐specific signaling under fed/insulin‐stimulated states will be necessary to clarify how these early systemic shifts relate to intramuscular signaling changes.

Taken together, the present findings indicate that a short‐term diet providing 40% of total energy from fat was associated with (i) selective visceral adipose expansion, (ii) a soleus‐specific reduction in Complex II abundance, and (iii) a fast‐twitch–biased reduction in ERK1/2 and Drp1 (Ser616) phosphorylation, without changes in total ERK1/2 or Drp1 protein levels and without changes in muscle mass. These findings suggest that short‐term high dietary fat intake may be associated with early remodeling of skeletal muscle signaling before overt changes in body or muscle mass become detectable. Although causal inference regarding ERK‐to‐Drp1 signaling cannot be made from the present design, the concordant directionality in EDL provides a focused hypothesis that can be tested experimentally using pathway perturbation (e.g., MEK/ERK modulation) and functional readouts of mitochondrial dynamics and muscle performance.

Several limitations of the present study should be acknowledged. First, the sample size was small (*n* = 5 per group), which may limit statistical power and increase sensitivity to inter‐individual variability. To address this, we emphasized effect sizes in addition to p values; nevertheless, findings showing borderline significance should be interpreted with caution. Second, the analyses were performed at a single time point following a 4‐week dietary intervention. Thus, the temporal sequence of signaling and mitochondrial adaptations—particularly the relationship between ERK1/2 and Drp1 phosphorylation—cannot be determined. Third, all measurements were obtained under fasting conditions, which may influence systemic metabolic parameters and muscle signaling pathways. Finally, functional assessments of mitochondrial respiration, mitochondrial morphology, and muscle contractile performance were not included, limiting direct interpretation of the physiological consequences of the observed molecular changes. Accordingly, the present findings should be interpreted as exploratory evidence of early molecular adaptation rather than definitive evidence of altered muscle physiology.

Future studies should aim to clarify the mechanistic and functional implications of the observed signaling changes. Time‐course experiments will be required to determine whether alterations in ERK1/2 phosphorylation precede changes in Drp1 phosphorylation or vice versa. In addition, experimental manipulation of ERK signaling, for example through pharmacological MEK/ERK inhibition or activation, would help test the proposed linkage between ERK activity and Drp1 Ser616 phosphorylation in skeletal muscle. Incorporating direct assessments of mitochondrial respiration and mitochondrial morphology, together with structural indices of fission/fusion state, will be essential to determine whether the observed signaling changes are accompanied by mitochondrial remodeling. Functional measurements—such as mitochondrial respiration, mitochondrial morphology, muscle fatigue resistance, and contractile properties—will be essential to establish whether the observed molecular adaptations translate into meaningful changes in muscle function. Finally, extending the dietary intervention period or comparing multiple fat contents may help delineate the transition from early adaptive signaling responses to maladaptive mitochondrial remodeling associated with prolonged high‐fat feeding.

In conclusion, the present study shows that a short‐term, physiologically relevant high‐fat diet (40% of total energy from fat) induces selective visceral adipose expansion and muscle‐type–specific molecular adaptations in skeletal muscle prior to overt changes in body weight or muscle mass. While mitochondrial fusion–related proteins and most OXPHOS components were preserved, a soleus‐specific reduction in Complex II and a fast‐twitch–biased decrease in ERK1/2 and Drp1 (Ser616) phosphorylation were observed without changes in total protein abundance. These findings suggest that short‐term high dietary fat intake is associated with muscle‐type–dependent changes in signaling activity before large‐scale mitochondrial protein remodeling occurs. Although causal relationships cannot be established from the present data, the coordinated changes observed in ERK and Drp1 phosphorylation in fast‐twitch muscle provide a focused framework for future mechanistic investigations into early dietary adaptation of skeletal muscle.

## AUTHOR CONTRIBUTIONS

**Arata Tsutaki:** Data curation; formal analysis; funding acquisition. **Hidetaka Imagita:** Supervision. **Asumi Yoshida:** Funding acquisition; methodology.

## CONFLICT OF INTEREST STATEMENT

The authors declare no conflicts of interest.

## Supporting information


**Figure S1.** Full blots corresponding to Figure 2 (Total OXPHOS).

**Figure S2.** Full blots corresponding to Figure 3 (Fusion‐related proteins).

**Figure S3.** Full blots corresponding to Figure 4 (Fission‐related proteins).

**Figure S4.** Full blots corresponding to Figure 5 (ERK‐related proteins).

## Data Availability

The data that support the findings of this study are available from the corresponding author upon reasonable request.
