# Supplementary material for: Short‐term high‐fat feeding induces muscle‐type–specific signaling adaptations in skeletal muscle of male rats
Source: Physiol Rep. 2026 Jun 15;14(12):e70904. doi: 10.14814/phy2.70904 (PMC13269180; doi:10.14814/phy2.70904)
Supplement: Supplementary file 1 — Figure S1. Full blots corresponding to Figure 2 (Total OXPHOS). [file PHY2-14-e70904-s004.pdf]

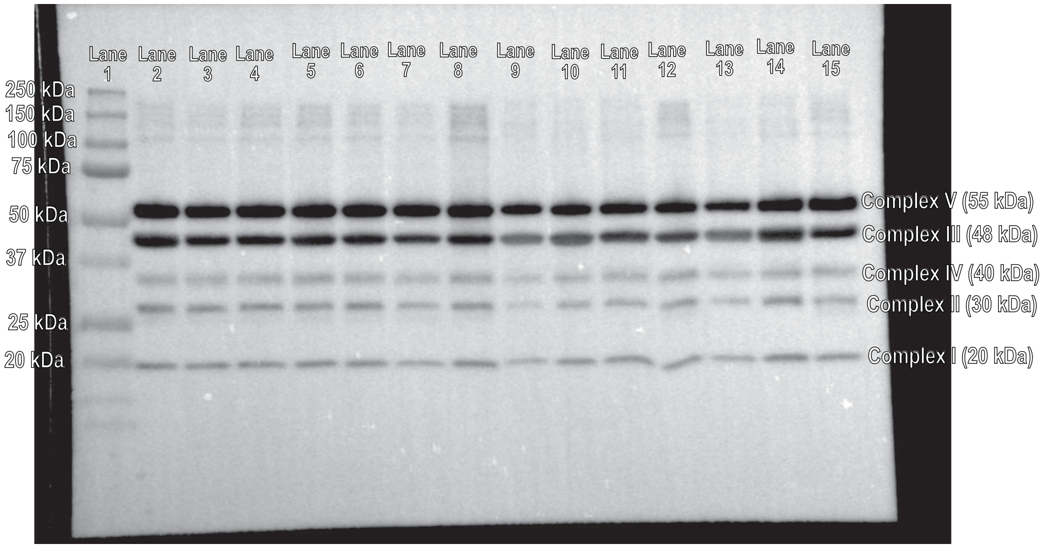

Lane	
1	Molecular weight marker
2	Empty
3	LFD #1 Soleus
4	Empty
5	HFD #1 Soleus
6	LFD #2 Soleus
7	Empty
8	HFD #2 Soleus
9	Reference sample
10	LFD #1 EDL
11	Empty
12	HFD #3 Soleus
13	LFD #2 EDL
14	Empty
15	HFD #4 Soleus

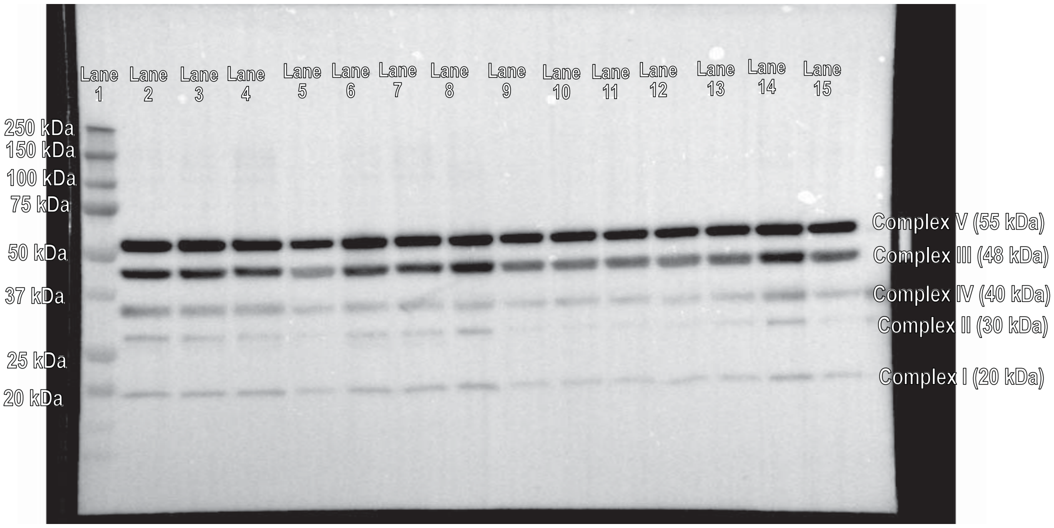

Lane	
1	Molecular weight marker
2	Empty
3	LFD #3 Soleus
4	Empty
5	HFD #1 EDL
6	LFD #4 Soleus
7	Empty
8	HFD #2 EDL
9	Reference sample
10	LFD #3 EDL
11	Empty
12	HFD #3 EDL
13	LFD #4 EDL
14	Empty
15	HFD #4 EDL

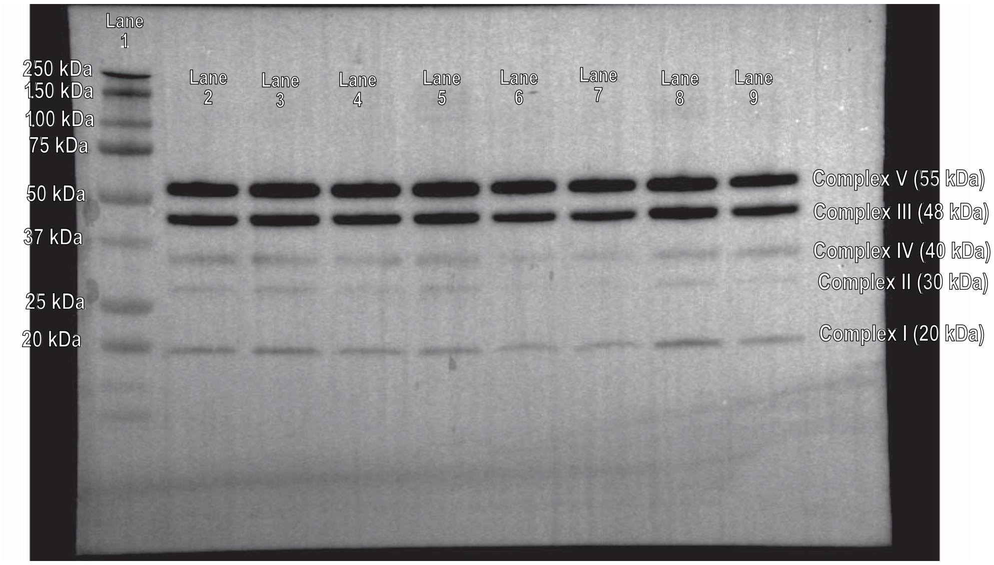

Lane	
1	Molecular weight marker
2	Empty
3	LFD #5 Soleus
4	Empty
5	HFD #5 Soleus
6	Reference sample
7	LFD #5 EDL
8	Empty
9	HFD #5 EDL

Supplementary Fig. S1. Full blots corresponding to Fig. 2 (Total OXPHOS)

Full, uncropped immunoblots of total OXPHOS corresponding to the representative blots shown in Fig. 2 are presented.
Molecular weight markers (kDa) are shown on the left of each blot, and the identities of OXPHOS complexes are indicated on the right.
Lane assignments for each membrane are provided in the tables adjacent to the blots.
A common reference sample was loaded on all membranes and used for inter-membrane normalization/alignment.
Empty lanes were included where applicable.
