# Supplementary material for: Short‐term high‐fat feeding induces muscle‐type–specific signaling adaptations in skeletal muscle of male rats
Source: Physiol Rep. 2026 Jun 15;14(12):e70904. doi: 10.14814/phy2.70904 (PMC13269180; doi:10.14814/phy2.70904)
Supplement: Supplementary file 2 — Figure S2. Full blots corresponding to Figure 3 (Fusion‐related proteins). [file PHY2-14-e70904-s002.pdf]

Mfn1

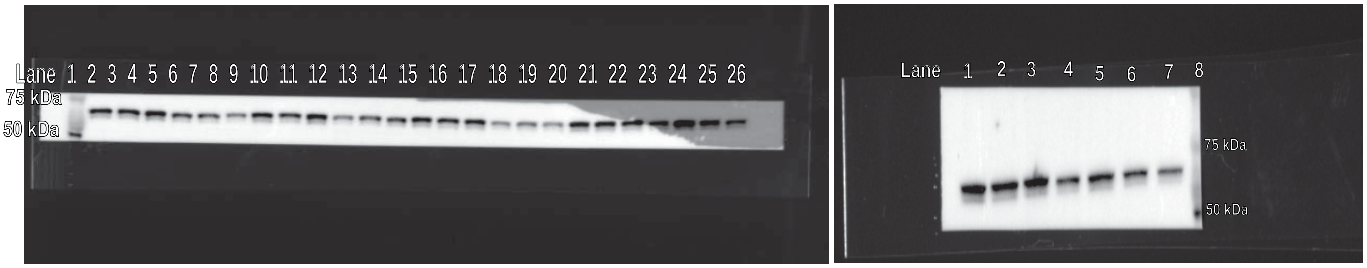

Lane assignment (left)																										
Lane	1	2	3	4	5	6	7	8	9	10	11	12	13	14	15	16	17	18	19	20	21	22	23	24	25	26
	Molecular weight marker	LFD #1 Soleus	Empty	HFD #1 Soleus	LFD #1 EDL	Empty	HFD #1 EDL	LFD #2 Soleus	Empty	HFD #2 Soleus	LFD #2 EDL	Empty	HFD #2 EDL	LFD #3 Soleus	Empty	HFD #3 Soleus	LFD #3 EDL	Empty	HFD #3 EDL	LFD #4 Soleus	Empty	HFD #4 Soleus	LFD #4 EDL	Empty	HFD #4 EDL	Reference sample

Lane assignment (right)								
Lane	1	2	3	4	5	6	7	8
	LFD #5 Soleus	Empty	HFD #5 Soleus	LFD #5 EDL	Empty	HFD #5 EDL	Reference sample	Molecular weight marker

Mfn2

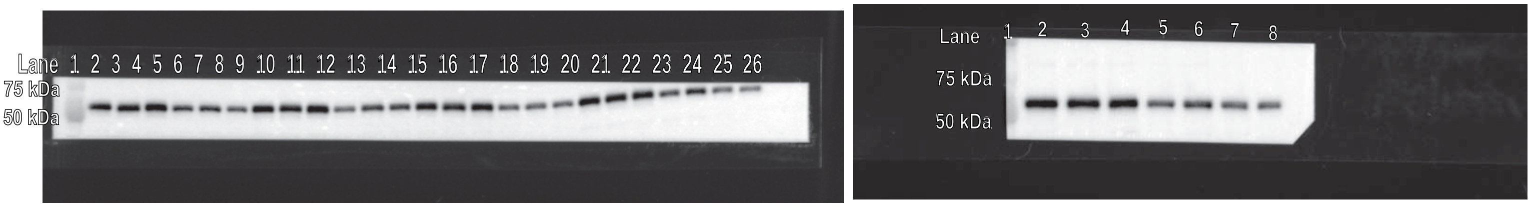

Lane assignment (left)																										
Lane	1	2	3	4	5	6	7	8	9	10	11	12	13	14	15	16	17	18	19	20	21	22	23	24	25	26
	Molecular weight marker	LFD #1 Soleus	Empty	HFD #1 Soleus	LFD #1 EDL	Empty	HFD #1 EDL	LFD #2 Soleus	Empty	HFD #2 Soleus	LFD #2 EDL	Empty	HFD #2 EDL	LFD #3 Soleus	Empty	HFD #3 Soleus	LFD #3 EDL	Empty	HFD #3 EDL	LFD #4 Soleus	Empty	HFD #4 Soleus	LFD #4 EDL	Empty	HFD #4 EDL	Reference sample

Lane assignment (right)								
Lane	1	2	3	4	5	6	7	8
	Molecular weight marker	LFD #5 Soleus	Empty	HFD #5 Soleus	LFD #5 EDL	Empty	HFD #5 EDL	Reference sample

Opa1

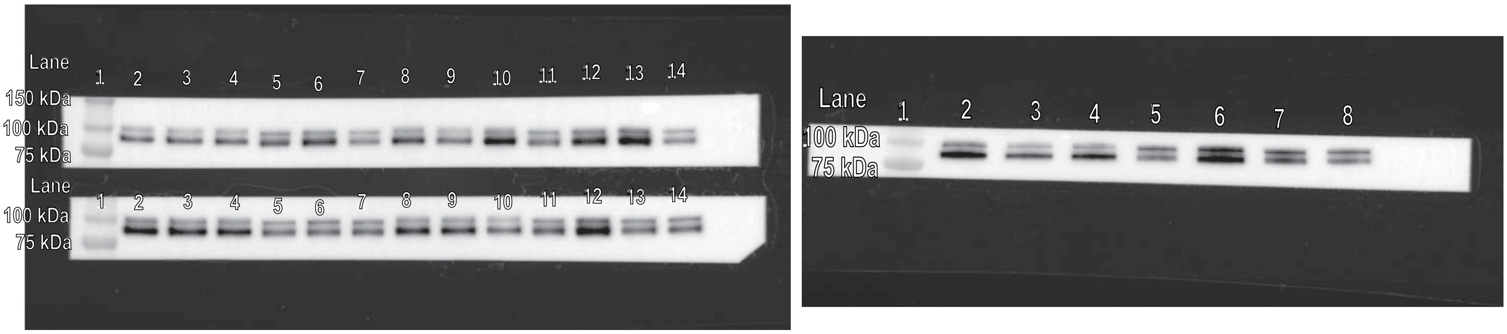

Lane assigment (left)														
Lane (Upper)	1	2	3	4	5	6	7	8	9	10	11	12	13	14
	Molecular weight marker	LFD #1 Soleus	Empty	HFD #1 Soleus	LFD #1 EDL	Empty	HFD #1 EDL	LFD #2 Soleus	Empty	HFD #2 Soleus	LFD #2 EDL	Empty	HFD #2 EDL	Reference sample
Lane (Lower)	1	2	3	4	5	6	7	8	9	10	11	12	13	14
	Molecular weight marker	LFD #3 Soleus	Empty	HFD #3 Soleus	LFD #3 EDL	Empty	HFD #3 EDL	LFD #4 Soleus	Empty	HFD #4 Soleus	LFD #4 EDL	Empty	HFD #4 EDL	Reference sample

Lane assignment (right)								
Lane	1	2	3	4	5	6	7	8
	Molecular weight marker	LFD #5 Soleus	Empty	HFD #5 Soleus	LFD #5 EDL	Empty	HFD #5 EDL	Reference sample

Supplementary Fig. S2. Full blots corresponding to Fig. 3 (Fusion-related proteins).

Full, uncropped immunoblots for Mfn1, Mfn2, and Opa1 corresponding to the representative blots shown in Fig. 3 are presented.
Molecular weight markers with corresponding molecular weight labels (kDa) are shown adjacent to each blot.
Lane assignments for each blot are provided in the tables adjacent to the images.
Empty lanes were included where applicable.
