# Supplementary material for: Short‐term high‐fat feeding induces muscle‐type–specific signaling adaptations in skeletal muscle of male rats
Source: Physiol Rep. 2026 Jun 15;14(12):e70904. doi: 10.14814/phy2.70904 (PMC13269180; doi:10.14814/phy2.70904)
Supplement: Supplementary file 3 — Figure S3. Full blots corresponding to Figure 4 (Fission‐related proteins). [file PHY2-14-e70904-s003.pdf]

p-Drp1 Ser 616

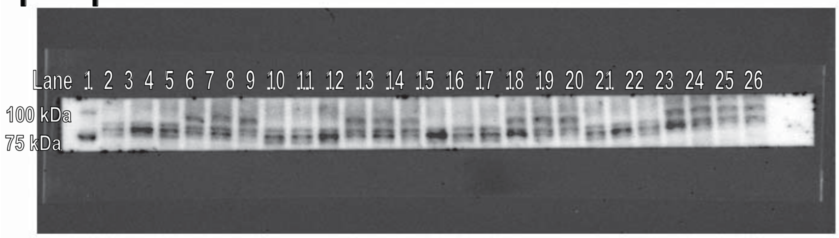

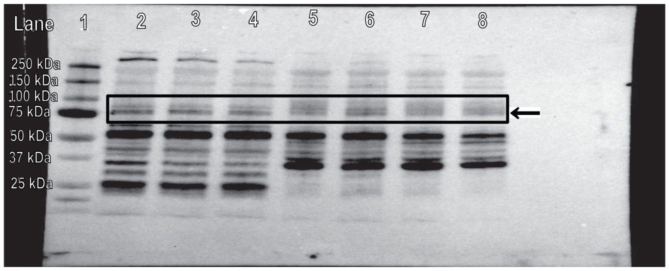

Lane assignment (left)																										
Lane	1	2	3	4	5	6	7	8	9	10	11	12	13	14	15	16	17	18	19	20	21	22	23	24	25	26
	Molecular weight marker	LFD #1 Soleus	Empty	HFD #1 Soleus	LFD #1 EDL	Empty	HFD #1 EDL	LFD #2 Soleus	Empty	HFD #2 Soleus	LFD #2 EDL	Empty	HFD #2 EDL	LFD #3 Soleus	Empty	HFD #3 Soleus	LFD #3 EDL	Empty	HFD #3 EDL	LFD #4 Soleus	Empty	HFD #4 Soleus	LFD #4 EDL	Empty	HFD #4 EDL	Reference sample

Lane assignment (right)								
Lane	1	2	3	4	5	6	7	8
	Molecular weight marker	LFD #5 Soleus	Empty	HFD #5 Soleus	LFD #5 EDL	Empty	HFD #5 EDL	Reference sample

Drp1

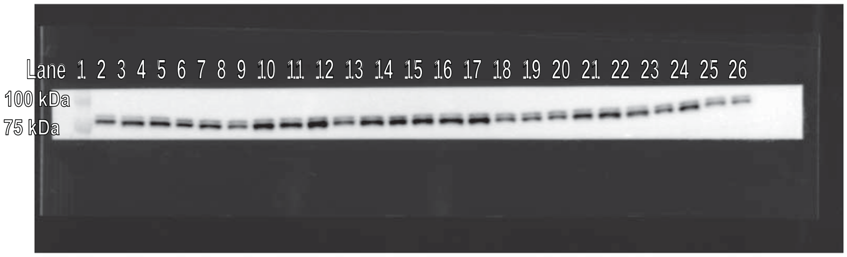

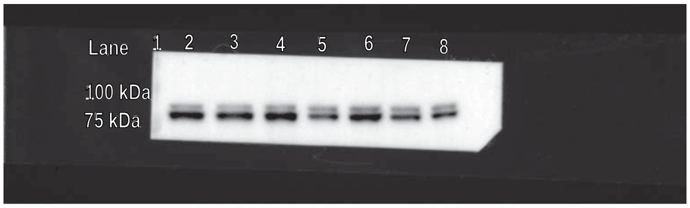

Lane assignment (left)																										
Lane	1	2	3	4	5	6	7	8	9	10	11	12	13	14	15	16	17	18	19	20	21	22	23	24	25	26
	Molecular weight marker	LFD #1 Soleus	Empty	HFD #1 Soleus	LFD #1 EDL	Empty	HFD #1 EDL	LFD #2 Soleus	Empty	HFD #2 Soleus	LFD #2 EDL	Empty	HFD #2 EDL	LFD #3 Soleus	Empty	HFD #3 Soleus	LFD #3 EDL	Empty	HFD #3 EDL	LFD #4 Soleus	Empty	HFD #4 Soleus	LFD #4 EDL	Empty	HFD #4 EDL	Reference sample

Lane assignment (right)								
Lane	1	2	3	4	5	6	7	8
	Molecular weight marker	LFD #5 Soleus	Empty	HFD #5 Soleus	LFD #5 EDL	Empty	HFD #5 EDL	Reference sample

Fis1

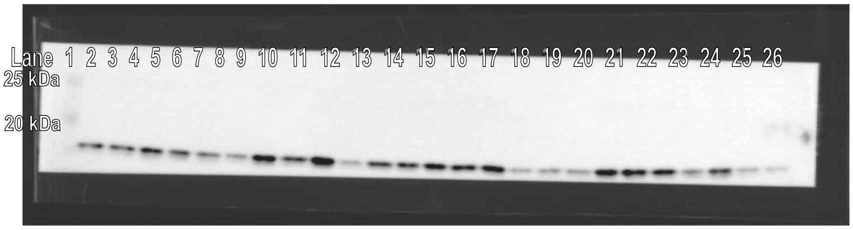

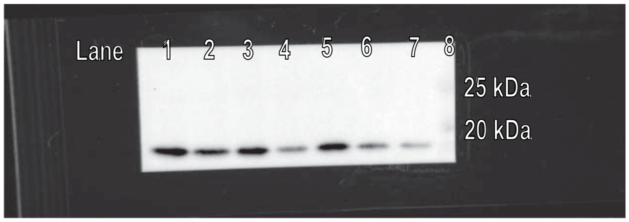

Lane assignment (left)																										
Lane	1	2	3	4	5	6	7	8	9	10	11	12	13	14	15	16	17	18	19	20	21	22	23	24	25	26
	Molecular weight marker	LFD #1 Soleus	Empty	HFD #1 Soleus	LFD #1 EDL	Empty	HFD #1 EDL	LFD #2 Soleus	Empty	HFD #2 Soleus	LFD #2 EDL	Empty	HFD #2 EDL	LFD #3 Soleus	Empty	HFD #3 Soleus	LFD #3 EDL	Empty	HFD #3 EDL	LFD #4 Soleus	Empty	HFD #4 Soleus	LFD #4 EDL	Empty	HFD #4 EDL	Reference sample

Lane assignment (right)								
Lane	1	2	3	4	5	6	7	8
	LFD #5 Soleus	Empty	HFD #5 Soleus	LFD #5 EDL	Empty	HFD #5 EDL	Reference sample	Molecular weight marker

Supplementary Fig. S3. Full blots corresponding to Fig. 4 (Fission-related proteins).

Full, uncropped immunoblots for p-Drp1 Ser616, Drp1, and Fis1 corresponding to the representative blots shown in Fig. 4 are presented.
Molecular weight markers with corresponding molecular weight labels (kDa) are shown adjacent to each blot.
Lane assignments for each blot are provided in the tables adjacent to the images.
Multiple non-specific bands were observed in the p-Drp1 Ser616 blot; quantification was performed for the band at the expected molecular weight, indicated by the arrow.
A common reference sample was loaded on all membranes and used for inter-membrane normalization/alignment.
Empty lanes were included where applicable.
